# The Next Frontier: Translational Development of Ubiquitination, SUMOylation, and NEDDylation in Cancer

**DOI:** 10.3390/ijms23073480

**Published:** 2022-03-23

**Authors:** Nicole E. Pellegrino, Arcan Guven, Kayleigh Gray, Punit Shah, Gargi Kasture, Maria-Dorothea Nastke, Anjan Thakurta, Stephane Gesta, Vivek K. Vishnudas, Niven R. Narain, Michael A. Kiebish

**Affiliations:** 1 BERG, 500 Old Connecticut Path, Framingham, MA 01701, USA; nicole.pellegrino@berghealth.com (N.E.P.); kayleigh.gray@berghealth.com (K.G.); punit.shah@berghealth.com (P.S.); gargi.kasture@berghealth.com (G.K.); maria.nastke@berghealth.com (M.-D.N.); stephane.gesta@berghealth.com (S.G.); vivek.vishnudas@berghealth.com (V.K.V.); niven.narain@berghealth.com (N.R.N.); michael.kiebish@berghealth.com (M.A.K.); 2 Oxford Centre for Translational Myeloma Research, University of Oxford, Oxford OX3 7LD, UK; athakurta@outlook.com; 3 Radcliffe Department of Medicine, University of Oxford, Oxford OX3 9DU, UK

**Keywords:** ubiquitination, NEDDylation, SUMOylation, post-translational modifications, cancer

## Abstract

Post-translational modifications of proteins ensure optimized cellular processes, including proteostasis, regulated signaling, cell survival, and stress adaptation to maintain a balanced homeostatic state. Abnormal post-translational modifications are associated with cellular dysfunction and the occurrence of life-threatening diseases, such as cancer and neurodegenerative diseases. Therefore, some of the frequently seen protein modifications have been used as disease markers, while others are targeted for developing specific therapies. The ubiquitin and ubiquitin-like post-translational modifiers, namely, small ubiquitin-like modifier (SUMO) and neuronal precursor cell-expressed developmentally down-regulated protein 8 (NEDD8), share several features, such as protein structures, enzymatic cascades mediating the conjugation process, and targeted amino acid residues. Alterations in the regulatory mechanisms lead to aberrations in biological processes during tumorigenesis, including the regulation of tumor metabolism, immunological modulation of the tumor microenvironment, and cancer stem cell stemness, besides many more. Novel insights into ubiquitin and ubiquitin-like pathways involved in cancer biology reveal a potential interplay between ubiquitination, SUMOylation, and NEDDylation. This review outlines the current understandings of the regulatory mechanisms and assay capabilities of ubiquitination, SUMOylation, and NEDDylation. It will further highlight the role of ubiquitination, SUMOylation, and NEDDylation in tumorigenesis.

## 1. Introduction

Post-translational modifications (PTMs) of proteins are covalent additions of biological moieties on specific amino acid residues post synthesis that control the abundance and function of proteins beyond their inherent transcriptional and translational regulation. PTMs are dynamically balanced by conjugation and de-conjugation by functionally opposing enzymes. They are crucial for adapting cells to various stress stimuli. The diverse group of PTMs comprises acetylation, phosphorylation, methylation, ubiquitination, SUMOylation, and NEDDylation, to name a few, and they affect virtually all cellular processes [1]. Ubiquitin, small ubiquitin-like modifier (SUMO), and neuronal precursor cell-expressed developmentally down-regulated protein 8 (NEDD8) are among the best-characterized PTMs. They are conjugated to thousands of proteins, modifying their function and fate in a highly dynamic manner. PTMs and the organized degradation of proteins are critical to cellular health, and dysregulation of these modifications has been implicated in a variety of cancers. Therefore, an emerging strategy to study PTM biology is to target protein modification processes and their regulation to induce cancer cell death. Structurally and functionally, the SUMO pathway bears relevance to the NEDD8 pathway because their constituents share highly conserved domain structures and mechanisms [2]. On the other hand, SUMO and NEDD8 pathways exhibit distinct functional features from the ubiquitin pathway that lead to different biological consequences [3].

In this review, alterations of ubiquitination, SUMOylation, and NEDDylation in health and cancer are discussed, with an emphasis on qualitative and quantitative analyses of these PTMs, which are potentially useful for therapeutic target identification and biomarker research.

## 2. Overview of Ubiquitination, SUMOylation, and NEDDylation

### 2.1. Ubiquitination

**Figure 1:**
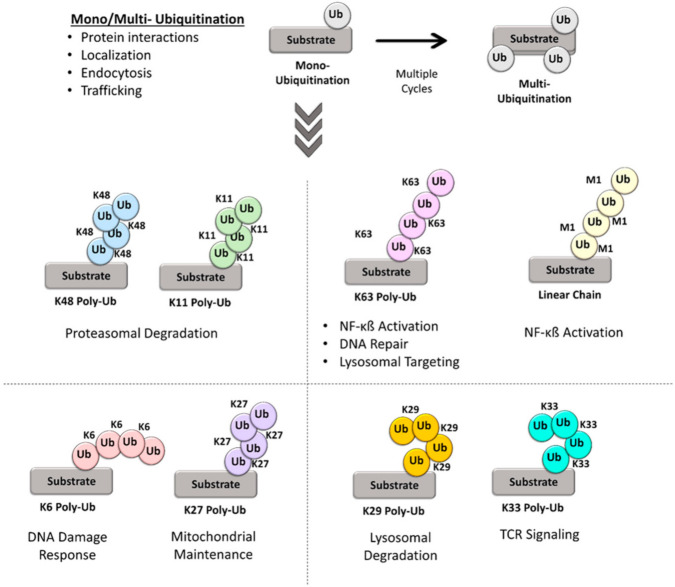
Schematic representation of the ubiquitin (Ub) architectures. Dynamic and complex ubiquitin architectures, ranging from monoubiquitination to multiple monoubiquitination to homotypic polyubiquitin linkages, that enable highly dynamic and complex regulation of cellular processes.

Ubiquitin is a highly conserved 76-amino acid protein (approximately 8 kDa), recognized as an intracellular signaling molecule that regulates virtually all aspects of eukaryotic biology. Ubiquitination is a crucial PTM in both normal homeostasis and in disease that involves the covalent attachment of ubiquitin to a target protein for proteasomal degradation or non-degradative signaling [4]. Ubiquitin can be conjugated to substrates as a monomer on one or more sites (most frequently lysines), referred to as monoubiquitination or multi-monoubiquitination, respectively. Ubiquitin can also be polymerized to form a polyubiquitin chain with different types of chain linkages [5]. Ubiquitin acts as a multifunctional signal due to its ability to form a variety of structures by virtue of its seven lysine residues (Lys6, Lys11, Lys27, Lys29, Lys33, Lys48, and Lys63), all of which can also be ubiquitinated to form isopeptide-linked ubiquitin chains that are recognized by distinct proteins. Another chain type, Met1-linked (‘linear’ chains), can also be generated when ubiquitin is attached to the N-terminus of a second ubiquitin. Although rare, mixed or branched polyubiquitin chains are also possible, and these profound architectures increase the complexity of the signals sent along several biological pathways [6]. The need for characterization of the relationship between these complex ubiquitin architectures and other key components (such as E1, E2, E3s, deubiquitinases (DUBs), and proteins that recognize ubiquitinated proteins by their ubiquitin-binding domains) has become very important. Ubiquitination is not only essential for protein degradation; ubiquitination at specific lysine residues also controls major physiological processes, including cell survival (DNA repair, cell cycle, protein turnover), differentiation, innate and adaptive immunity (inflammatory signaling and stress response), endocytosis, protein–protein interactions, transcriptional regulation, and intracellular trafficking, to cite only a few [7]. For example, the fate of a ubiquitin-linked protein, such as its cellular localization and protein activity (promoting or interfering with protein interactions), is dependent on lysine chain-specific polyubiquitination [8] (Figure 1). Similarly, Lys48-linked polyubiquitin chains usually target proteins for degradation, while Lys63-linked polyubiquitination has been shown to be involved in the regulation of NF-kB signaling, Lys27-linked polyubiquitin chains to play a role in the regulation of mitophagy, and Lys11-linked polyubiquitination to contribute to the degradation of cyclins in the control of the cell cycle [5].

**Figure 2:**
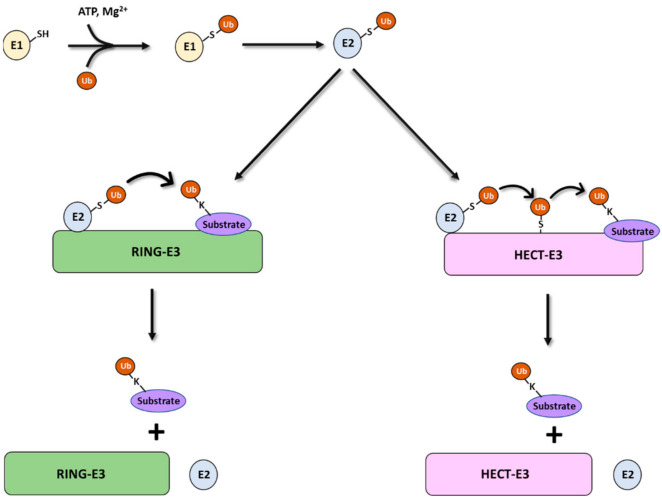
Schematic representation of the three-step enzymatic cascade for ubiquitination of substrates. Depending on the E3 ubiquitin ligase involved, ubiquitin linked to the E2 ubiquitin-conjugating enzyme can be transferred and subsequently conjugated to the substrate protein by at least two mechanisms.

Protein ubiquitination occurs in a three-step process involving a ubiquitin activating enzyme (E1), a conjugating enzyme (E2), and a ubiquitin-protein ligase (E3) (Figure 2). Initially, a single ubiquitin moiety is attached to an active site cysteine residue within the E1 through a thioester bond in an ATP-dependent manner. The activated ubiquitin is then trans-thiolated from the E1 to the active site cysteine of an E2. In the last step, in most cases, ubiquitin is then transferred from the E2 to the ε-amino group of a substrate in an E3-dependent manner [9]. E3 ligases use domains dedicated to the recruitment of specific substrates, while they determine the linkage specificity of chain formation dependent on their catalytic module. The E3 ligases that belong to the really interesting new gene (RING) domain family mediate the direct transfer of ubiquitin from E2 to substrate [10]. For the E3s of the homologous to E6AP carboxyl terminus (HECT) domain family, a thioester intermediate is involved which contains an active site cysteine charged with ubiquitin to directly select the ubiquitin residue for chain elongation (Figure 2) [11].

The ubiquitin–proteasome system (UPS), a network of E3 ligases and DUBs, is the primary proteolytic quality control system in cells. UPS mediates the removal of misfolded and aggregated proteins as well as the targeted degradation of most normal proteins that are no longer needed. Most proteins that are critical for cellular regulations and functions are targets of the UPS, and their rapid and timely degradation is essential in regulating cellular processes [12]. UPS controls the abundance of pro- or anti-apoptotic proteins and dictates cell survival versus death to regulate apoptosis. The function of E3 ligases can be reversed by DUBs removing ubiquitin from substrate proteins or remodeling ubiquitin chains on target proteins. This is a critical process for almost all cellular signaling pathways, such as the cell cycle, apoptosis, receptor downregulation, and gene transcription. DUBs belong to the family of cysteine proteases and cleave the isopeptide or peptide bond with high specificity [13]. Considering the significance of the UPS in regulating the wide array of cellular processes, dysregulation of the UPS has been associated with the disruption of cellular homeostasis.

### 2.2. SUMOylation

**Figure 3:**
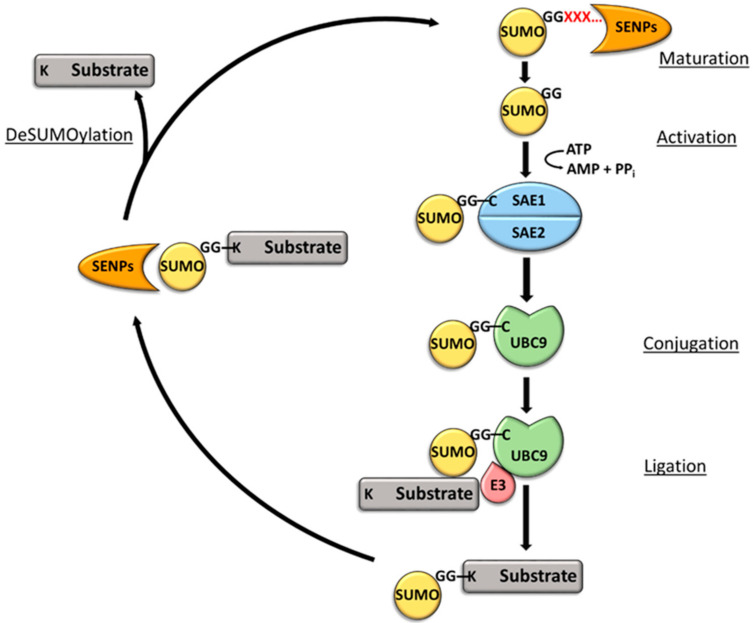
The SUMOylation catalytic cycle. SENP family protease removes C-terminal amino acids of the SUMO precursor. The mature SUMO is activated in the ATP-dependent formation of a thioester bond with the SAE2 subunit of the E1 activating enzyme. SUMO is transferred to the E2 conjugating enzyme, UBC9, by a trans-thiolation reaction. An E3 ligase facilitates the ligation of SUMO to the substrate by binding to both UBC9 and the substrate. SUMOylation is reversed when a protease from the SENP family cleaves the SUMO modification.

SUMO has a similar three-dimensional structure to ubiquitin [14], although the proteins share less than 20% amino acid sequence identity [15]. Five SUMO paralogues, (SUMO1-5), each approximately 11 kDa, are considered to be functionally significant in mammalian systems [16]. SUMO2 and SUMO3 are more than 95% identical to each other, and SUMO-1 shares 48% and 46% amino acid sequence identity with SUMO-2 and SUMO-3, respectively [17]. Unlike the ubiquitination process, the SUMOylation pathway relies on a single E2 enzyme, ubiquitin-conjugating enzyme 9 (UBC9). Several SUMO E3 ligases have been identified that promote the transfer of SUMO from E2 to specific substrates. Although not essential, SUMO E3 ligases may be important in regulating substrate selection, particularly for substrates that lack consensus SUMO acceptor motifs. While monoSUMOylation is the most commonly observed modification, assembly of polySUMO or mixed ubiquitin–SUMO chains are possible [18]. SUMOylation events mainly occur at a lysine within the consensus motif ΨKxD/E (where Ψ represents a large hydrophobic amino acid and x represents any amino acid) [16,19]. Upon translation of the SUMO precursor, several amino acids are removed from its C-terminus by sentrin-specific protease (SENP) family proteases [16], exposing a di-glycine motif (Figure 3). The SUMO protein is then activated by the ATP-dependent formation of a thioester bond between the C-terminal glycine residue and a cysteine residue in the active site of SUMO-activating enzyme subunit 2 (SAE2), a heterodimer of the E1 activating enzyme comprised of SAE1 and SAE2. SUMO is transferred to the active site cysteine of the E2 conjugating enzyme, UBC9, via a thioester bond. UBC9 then catalyzes the formation of an isopeptide bond between the C-terminal glycine of SUMO and the ε-amino group of a substrate lysine. This is sometimes aided by an E3 ligase, which binds to both the E2 enzyme and the substrate. Following that, the SENP family of proteases deSUMOylates the substrates [16,20].

Consequences for the SUMOylated substrate include inhibition of interaction by blockage of protein-interaction sites, promotion of interaction by formation of new protein-binding faces, and alteration of activity or exposition of previously occluded binding sites due to changes in conformation [16]. SUMOylated substrates can non-covalently interact with proteins that contain a SUMO-interacting motif (SIM) with a common hydrophobic consensus of ψψxψ (where ψ most commonly represents isoleucine, leucine, or valine and x represents any amino acid) [21].

### 2.3. NEDDylation

**Figure 4:**
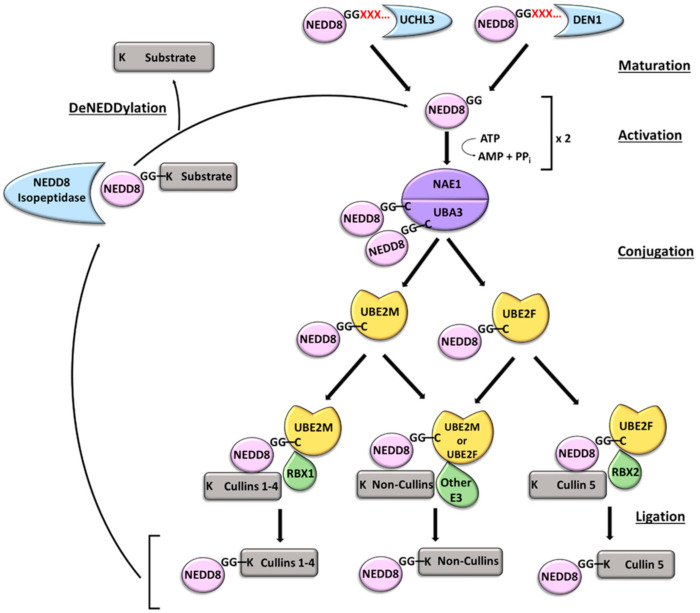
The NEDDylation catalytic cycle. A hydrolase, UCHL3 or DEN1, removes C-terminal amino acids of the NEDD8 precursor. The mature NEDD8 is activated in the ATP-dependent formation of a thioester bond with the UBA3 subunit of the E1 activating enzyme NAE. NEDD8 is transferred to an E2 conjugating enzyme, UBE2M or UBE2F, by a trans-thiolation reaction. A substrate specific E3 ligase facilitates ligation of NEDD8 to the substrate by binding to both the E2 enzyme and substrate. NEDDylation is reversed when a NEDD8 isopeptidase cleaves the NEDD8 modification.

NEDD8, an 81-amino acid protein (approximately 9 kDa), is closely related to ubiquitin, as the two proteins are 60% identical and 80% homologous [22]. The amino acids beyond Gly76 are cleaved by either the C12 family peptidase ubiquitin C-terminal hydrolase L3 (UCHL3), which also cleaves C-terminal extensions of ubiquitin [23], or the NEDD8-specific C48 family peptidase deNEDDylase 1 (DEN1/NEDP1/SENP8) [24,25] before NEDD8 can attach to a substrate [26] (Figure 4). NEDD8 is then adenylated by the heterodimer E1 NEDD8-activating enzyme (NAE) that consists of NAE1 (APPBP1) and ubiquitin-like modifier activating enzyme 3 (UBA3) [27], and is transferred to a specific cysteine of NAE to form a thioester [28]. A trans-thiolation reaction transfers NEDD8 to one of two E2 conjugating enzymes, ubiquitin-conjugating enzyme E2 M (UBE2M/UBC12) or ubiquitin-conjugating enzyme E2 F (UBE2F) [29]. Following that, a substrate-specific E3 ligase facilitates the conjugation of NEDD8 carboxy-terminal glycine, Gly76, to a lysine residue of the substrate by an isopeptide bond [26,30]. This conjugation can lead to the formation of mono-NEDD8, poly-NEDD8, or mixed ubiquitin–NEDD8 chains [31,32]. In most cases, NEDD8 binding is covalent, but there have been some reports of non-covalent interactions [33]. NEDDylation modifies the three-dimensional surfaces of substrates by inducing conformational changes that alter protein activity, creating new binding sites, or blocking protein–protein interactions or other post-translational modifications [26].

Cullins, subunits of cullin-RING E3 ubiquitin ligases (CRLs), are the best characterized NEDDylation substrates [30]. CRLs are the largest family of E3 ubiquitin ligases, and NEDDylation of CRLs stimulates their activity [34]. UBE2M uses the E3 RING box protein 1 (RBX1) and defective in cullin NEDDylation 1 (DCN1) in NEDDylation of cullins 1–4, while UBE2F uses RBX2 for cullin 5 attachment [35,36]. There are also non-cullin NEDDylation substrates (e.g., tumor protein P53 (p53), E3 ubiquitin-protein ligase (MDM2), breast cancer-associated gene 3 (BCA3), and transforming growth factor beta-2 (TGF-β II)). These proteins are involved in cell cycle regulation, transcriptional activity, tumor suppression, and DNA repair [37,38,39]. Substrate deNEDDylation occurs via NEDD8 isopeptidases, including COP9 signalosome subunit 5 (CSN) for cullins, NEDP1 [23], and proteases with dual specificity for NEDD8 and ubiquitin [30].

## 3. Ubiquitination, SUMOylation, and NEDDylation in Cancer

Post-translational modifications by ubiquitin and ubiquitin-like proteins SUMO or NEDD8 are described in a multitude of cellular processes and are essential in cellular pathways and functions that are often dysregulated in cancer. Moreover, the enzymes of these post-translational modification pathways have been shown to be often abnormally regulated in many tumor types [40,41]. The following section will focus on oncogenic and tumor-suppressor pathways that are controlled by ubiquitin or ubiquitin-like protein modifications.

The ubiquitin proteasome system (UPS) is essential in orchestrating protein degradation and in the fundamental regulation of cellular processes [42]. Hence, divergent regulatory mechanisms that affect genetic alteration, abnormal expression, or dysfunction of the UPS can subsequently induce human cancer pathogenesis. Both tumor-suppressing and -promoting pathways have elements that are tightly regulated by the ubiquitination process. Ubiquitin system components with cancer-related roles include E3 ligases, E1 and E2 enzymes, DUBs, and the proteasome. Members of the ubiquitin pathway have been reported to be mutated or overexpressed in various cancers and are associated with human malignancies by regulating the activity or degradation of tumor-promoting or tumor-suppressor proteins.

E3 ligases are one of the most prevalent cancer-related functional gene families [43]. E3 ligases regulate diverse cellular processes, such as cell proliferation, cell cycle arrest, and apoptosis. Mutations or dysregulation of the expression of E3 ligases, which are key players in the UPS, clinically correlate with poor survival and prognosis [44]. E3 ubiquitin ligases can be either tumor suppressors or oncoproteins, depending on their ability to trigger degradation of either oncoproteins or tumor-suppressor proteins, respectively. One of the most prominent examples of a tumor-suppressor protein is p53, a transcription factor. The cell protective function of p53 is often lost in cancer. Cellular functions of p53 include regulation of cell cycle and death, autophagy, DNA damage repair, senescence, and metabolism. TP53 is the most commonly mutated gene in human cancers and its mutant protein facilitates cancer progression [45]. The main negative regulator of p53 is the murine double minute 2 (MDM2) oncoprotein. MDM2 is a p53-specific E3 ubiquitin ligase acting as a cellular antagonist of p53 by restricting the p53 growth-suppressive function in p53 wild-type cells. In basal conditions, MDM2 and p53 regulate each other through an autoregulatory loop. Activation of p53 transcribes MDM2 mRNA and increases MDM2 protein expression, which, in turn, inhibits p53 activity by three mechanisms: (i) upon binding, MDM2 ubiquitinates p53 through its E3 ligase activity, leading to K48-linked chain formation and subsequent degradation of p53 by the ubiquitin proteasome system; (ii) p53 binding to its targeted DNA is blocked by the interaction of MDM2 with p53, which subsequently makes p53 ineffective as a transcription factor; and (iii) MDM2 promotes export of p53 out of the cell nucleus, which renders p53 inaccessible to its targeted DNA and further reduces its transcriptional ability [46,47]. While being an efficient inhibitor of the tumor-suppressive functions of p53, MDM2 is oncogenic when overexpressed [48]. A high frequency of MDM2 gene amplifications leading to overexpression has been identified in a variety of tumor types, including breast carcinomas [49], glioblastomas [50], B cell lymphomas [51], and myeloid neoplasms [52]. Additionally, MDM2 overexpression can be the consequence of a variety of other mechanisms, such as single-nucleotide polymorphism, enhanced transcription, or increased translation [53,54]. A clear correlation of MDM2 overexpression with poor clinical prognosis and poor response to cancer therapies has been demonstrated over the past decades [53,55]. Mutations in breast cancer type 1 susceptibility protein (BRCA1) and its heterodimeric binding partner BRCA1-associated RING domain protein 1 (BARD1) confer a high risk for the development of breast cancer. The BRCA1–BARD1 heterodimeric RING finger complex serves as a central regulator and guardian of genomic integrity [56]. The BRCA1–BARD1 complex exhibits ubiquitin ligase activity and the distinct types of ubiquitin additions by the BRCA1–BARD1 complex on substrate proteins facilitates its critical roles in DNA double-stranded break repair, cell-cycle regulation, and transcriptional regulation. Its role in error-free repair of DNA double-strand breaks is important for its tumor-suppression activity. Breast-cancer derived mutations in both subunits of the BRCA1–BARD1 complex lead to inactivation of its ligase activity [57,58]. It is well established that the ligase activity is required for BRCA1–BARD1’s role in the maintenance of genome integrity and transcriptional regulation; however, its E3 ligase activity has not been directly linked to its role as a tumor suppressor [59]. Ubiquitination on BRCA1–BARD1, on the substrate, or on both may affect the ligase activity of the complex. BRCA1–BARD1 is largely localized to the nucleus and facilitates DNA damage-induced mono-ubiquitination of nucleosome histones. Following that, BRCA1–BARD1 catalyzes the formation of multiple polyubiquitin chains on itself, which significantly stimulates its ligase activity [60]. Ubiquitination appears to play an important role in regulating the location and activity of BRCA1–BARD1, and disruption of histone ubiquitylation leads to defects in mitotic cell growth, meiosis, and DNA repair [61].

E1 activating enzymes initiate more than a dozen post-translational modifications that influence a plethora of cellular substrates. Subsequently, the functional diversity and breadth of these modifications position E1 enzymes in orchestrating roles in almost every aspect of cancer cell biology [62]. During tumorigenesis, cells are remodeled by the accumulation of genetic aberrations and other alterations, which promotes tumor cell proliferation and allows them to evade growth suppressors [63]. Post-translational modifications initiated by E1 activating enzymes are described in these alteration processes of cancer cell signaling through proteolytic and non-proteolytic mechanisms [64]. For example, several growth factor receptors, such as epidermal growth factor receptor (EGFR), MET proto-oncogene (MET), platelet-derived growth factor receptor (PDGFR), and their downstream kinases are regulated by ubiquitination and resulting proteasomal degradation in normal conditions. Changes driven by tumorigenesis will decrease the degradation of these proteins due to their stabilization which will subsequently result in mitogenic signaling. Such an example is presented by the enhancement of the function of several ubiquitin E3 ligases by the initiation of ubiquitination through E1 activating enzymes UBA1 and UBA6. Conversely, it has been shown that the anti-tumor activity of several tumor-suppressive proteins is reduced by alterations that enhance their ubiquitin-dependent proteasomal degradation [65]. These insights become particularly relevant in the development of therapeutic inhibitors, where alterations or inhibitions of E1 enzymes may lead to further activation of mitogenic signaling, resulting in unfavorable outcomes, or, on the contrary, may lead to stabilization of tumor suppressors, hence slowing tumor progression.

The family of E2 conjugating enzymes are central players in the enzymatic cascade of protein ubiquitination that couple activation of the ubiquitin or ubiquitin-like proteins to conjugation events downstream. The family of E2 conjugating enzymes is comprised of 40 members known to modulate the stability of proteins and ubiquitination through the conjugation of ubiquitin to target proteins [66]. E2 conjugating enzymes have been proposed to be critical factors in carcinogenesis, in which they are often dysregulated and can contribute to various tumor-relevant processes. Several E2s have been shown to drive cell cycle progression, apoptosis, DNA repair, and oncogenic signaling pathways during tumorigenesis. Multiple studies have shown altered expression of E2 enzymes and their association with poor survival in several cancers, such as breast, pancreatic, gastric, colon, cervix, bladder, lung, and multiple myeloma [67,68,69,70].

The wide functional diversity of DUBs has an important impact on the regulation of multiple biological processes, such as cell cycle control, DNA repair, chromatin remodeling, and several signaling pathways that are frequently altered in cancer [71]. DUBs display critical roles in different steps of cancer progression, such as epithelial-to-mesenchymal transition, cell migration, and in the regulation of apoptotic processes, either promoting (USP2, USP7, USP8, USP9X, USP15, USP16, USP17, USP28, USP41, CYLD, UCHL1, A20, and ATXN3) or suppressing apoptosis (USP2, USP9X, USP18, UCHL3, and A20) [72]. The ability to advance through different stages of the cell cycle regardless of inhibitory signals is one of the hallmarks of cancer [63,73]. Several DUBs have been found to play roles in cell cycle control of cancers via the regulation of different cell cycle checkpoints: G1 phase control (e.g., USP3, USP10), G1/S transition (e.g., ubiquitin carboxyl-terminal hydrolase BAP1 (BAP1)), S/G2 transition (e.g., DUB3), and mitotic phase (e.g., USP7). The cell cycle checkpoints are controlled by cyclins and cyclin-dependent kinases (CDKs) [74]. DUBs have been shown to interact with the cyclin–CDK complex. The depletion of DUBs increases the levels of CDK inhibitors at different cell cycle phases and results in cell cycle arrest. In addition, DUBs could regulate transcription factors for cell cycle control, which then promotes cell proliferation by inhibiting the expression of the cell cycle inhibitor p27 [75]. This example indicates the possible regulatory role of DUBs in cancer proliferation. DUBs have also been reported to regulate cell proliferation through different cell signaling pathways, such as Wnt/β-catenin signaling, p53-mouse double minute 2 (MDM2) signaling, PI3K–AKT signaling, AR signaling, and transforming growth factor beta (TGF-β) signaling. Aberrant canonical Wnt/β-catenin signaling is tightly associated with many solid and liquid tumors [76]. From a different angle, suppression of DUBs (e.g., USP2) leads to MDM2 destabilization, resulting in p53 activation and thus cancer cell survival.

Cancer cells have the ability to alter their metabolism in order to support the nutrient and energy requirements for cell survival and growth [77]. Ubiquitination and deubiquitination are involved in the regulation of cancer metabolism. Multiple signaling pathways, transcription factors, and metabolic enzymes participate in the modulation of cancer metabolism. The ubiquitination of proteins, such as Ras-related GTP-binding protein A (RagA), mammalian target of rapamycin (mTOR), Phosphatase and tensin homolog (PTEN), Protein kinase B (PKB, also known as Akt), c-Myc, and p53, play an important role in regulating the activity of the mTOR Complex 1 (mTORC1), 5’ AMP-activated protein kinase (AMPK), and PTEN–AKT signaling pathways. Additionally, when cells are in a state of limited energy intake or starvation, transcription factors can activate the related genes in glycolysis and the tricarboxylic acid cycle, increase hepatic glucose production, reduce insulin secretion, and provide a substrate for gluconeogenesis. Among them, hypoxia-inducible factor-1α (HIF- 1α), c-Myc, and p53 are closely related to cell metabolism [78].

Ubiquitination has been reported to play a critical role in host cell defense and immunological tumor microenvironment modulation by regulating cell signal transduction pathways [79,80]. In this regard, ubiquitination in one aspect can regulate processes of the immune response temporally and spatially [81], but in another aspect ubiquitination can effectively induce anticancer immunity by regulating the degradation of signal transduction molecules to preserve the balance between tumor suppressors and oncoproteins [82,83]. One of the major pathways regulated is the NF-κB signaling pathway. NF-κB, a transcription factor pivotal for inflammatory responses, is one of the most important molecules that links chronic inflammation with tumorigenesis. Ubiquitination plays an important role in the process of NF-κB activation. NF-κB is retained in an inactive state in the cytosol, where its transcriptional activity is kept in check by the inhibitor of NF-κB alpha (IκBα) [84]. However, upon phosphorylation by IκB kinases (IKKs) and subsequent polyubiquitination, IκBα undergoes ubiquitin-dependent proteasomal degradation through E3 ligase β-TrCP removing the inhibitory activity and allowing nuclear translocation and transcriptional activation of NF-κB [85].

The ability to evade apoptosis is one of the essential changes in cancer cells that causes malignant transformation. The two extrinsic and intrinsic pathways in apoptosis both involve the activation of caspase molecules. DUBs were found to target different pro- and anti-apoptotic proteins in both the extrinsic and intrinsic pathways in normal conditions. Depletion of DUBs controls apoptotic responsiveness in tumor necrosis factor alpha apoptosis-inducing ligand (TRAIL)-resistant tumor cells [86]. In addition, knockdown of DUBs leads to apoptosis in multiple myeloma cells [87]. On the other hand, many DUBs have been reported to bind with and stabilize oncoproteins, such as c-MYC. USP22 promotes the deubiquitination of c-MYC in breast cancer cells, resulting in increased levels of c-MYC. Overexpression of USP22 stimulates tumorigenic activity in breast cancer cells and is closely correlated with breast cancer progression [88].

The ubiquitin system has been a major focus in the pursuit of novel therapeutics targeting this molecular mechanism. There are several potential strategies that could be deployed to target the three enzymatic steps of the ubiquitin pathway. Broad targeting of E1, E2, and the proteasome are possible, but targeting the E3 enzymes or substrate binding site is the most straightforward strategy because it offers enhanced specificity. Furthermore, targeting USPs, a highly specialized class of DUBs, has emerged as a translational novel anticancer therapy. Targeting E1 and inhibiting the first stage of the ubiquitin pathway is therapeutically very risky due to lack of specificity (only two E1 enzymes). There are only a few E1 inhibitors that have been reported [89,90,91]. Among these, MLN7243 is reported to be a UBA1 inhibitor and is currently in Phase I clinical trials in adult participants with advanced solid tumors (ClinicalTrials.gov identifiers: NCT02045095) [92]. Alternatively, blocking the protein–protein interactions (PPI) between E2s and E3s or, similarly, E3s and their substrates has been an effective strategy since this approach offers direct targeting of the interfaces of ubiquitination complexes [93]. However, PPI inhibition has so far been considered challenging because it is difficult to design small molecules that can bind to the flat and highly hydrophobic interfaces of PPIs. In recent years, new PPI stabilizers and inhibitors, including small molecules, peptides, and antibodies, have entered clinical trials [94]. Proteasome inhibition is another therapeutic approach that proved to be successful to overcome chemoresistance by NF-κB inhibition-induced chemosensitization. Clinical trials using the currently approved proteasome inhibitors (bortezomib, carfilzomib, and ixazomib) have transformed the treatment of multiple myeloma by establishing new standards of care [95]. However, because of its vital role in cellular homeostasis, patients do experience some level of toxicity [96,97]. DUB inhibitors represent a promising class of anticancer therapeutics due to their regulatory mechanisms and significant roles in the progression of various types of cancer [98]. The development of selective DUB inhibitors has been limited because of insufficient understanding of DUB biology and difficulties in establishing robust biochemical assays for compound screening as well as cellular and in vivo models suitable to investigate DUB inhibition [99]. A large number of small-molecule DUB inhibitors targeting USPs have been reported but only a very limited number of inhibitors have made their way to preclinical or clinical phases, such as the USP30 inhibitor (preclinical phase) and VLX1570 (Phase I/II).

SUMOylation and key proteins in the SUMO pathway have been implicated in several different types of cancers. SUMOylation can either lead to or prevent cell proliferation and carcinogenesis depending on the substrate. P53 is stabilized by SUMOylation, which inhibits cell proliferation and induces tumor cell death or senescence [100]. AKT SUMOylation, however, promotes cell proliferation and tumorigenesis [101].

In breast cancer, SUMOylation plays a key role. Amplified-in-breast cancer 1 (AIB1) is overexpressed in breast cancer and its SUMO-1 modification likely regulates its transcriptional activity [102]. Increased SUMOylation of cellular substrates stimulates tumorigenesis [103], and SUMOylation of zinc finger protein 131 (ZNF131) reduces estrogen-induced cell growth by decreasing estrogen signaling [104]. SUMOylation of breast cancer type 1 (BRCA1) after genotoxic stress is involved in DNA damage repair, and BRCA1 mutations are linked to a high risk of breast cancer and ovarian cancer [105]. SUMO protease dynamics also affect breast cancer. Sentrin-specific protease 1 (SENP1) can amplify invasion and lung metastasis of triple negative breast cancer cells [106]. Upregulation of peptidyl-prolyl cis-trans isomerase NIMA-interacting 1 (PIN1), which is stabilized by SENP1-mediated deSUMOylation, promotes oncogenesis [107]. Sentrin-specific protease 5 (*SENP5*) silencing inhibits anchorage-independence growth, proliferation, migration, and invasion [16]. Long transcript of two sentrin/small ubiquitin-like modifier (SUMO)-specific protease 7 (SENP7L) stimulates aberrant proliferation [108]. Sentrin-specific protease 6 (*SENP6*) mRNA expression is lower in breast cancer tissue than in normal tissue [109]. The SUMO-activating enzyme subunit ½ (SAE1/SAE2) may promote metastasis in MYC-driven breast cancer [110], and the SUMO-conjugating E2 enzyme UBC9 has also been shown to advance metastasis in breast cancer [111].

The members of the SUMO pathway are also significantly involved In prostate cancer. Inhibition of human ubiquitin specific peptidase 39 (USP39) SUMOylation promotes the USP39-enhanced proliferation of prostate cancer cells [112], and the inhibition of methyl methane sulfonate ultraviolet sensitive gene clone 81 (MUS81) SUMOylation can contribute to tumorigenesis [113]. SENP1 is linked to prostate cancer progression because SENP1 regulation of matrix metallopeptidase 2 (MMP2) and matrix metallopeptidase 9 (MMP9) through the hypoxia-inducible factor 1-alpha (HIF-1α) signaling pathway promotes progression [114]. The SUMO E3 ligase PIAS1 interacts with the androgen receptor and FOXA1 at chromatin sites, which leads to an increase in prostate cancer cell proliferation [115].

The SUMOylation pathway has important roles in colorectal cancer. These include the SUMOylation of prolyl hydroxylase 3 (PHD3), decreasing HIF-1-induced migration and invasion of the cancer cells [116,117], and the oncogenic function of tripartite motif-containing 24 (TRIM24), a SUMO E3 ligase [118]. In multiple myeloma, SUMOylation of β-catenin leads to the dysregulation of proliferation [119], and the inhibition of SUMO protease SENP1 induces apoptosis and reduces proliferation by inactivating IL-6-induced NF-κB signaling [120]. The suppression of UBC9 by miR-214 overexpression inhibits the proliferation of glioma cells [121], and the stabilization of cyclin-dependent kinase 6 (CDK6) by SUMO-1 modification drives the cell cycle for glioblastoma development and progression [122]. SUMOylation and members of the SUMO pathways are involved in many other cancers, such as lung cancer [123], leukemia [124], melanoma [125], gastric cancer [126], bladder cancer [127], osteosarcoma [128], hepatocellular carcinoma [129], oral squamous cell carcinoma [130], and pancreatic ductal adenocarcinoma [131,132], and can be used as potential therapeutic targets for cancer.

SUMOylation proteins facilitate cell cycle progression, and inhibition of the cascade proteins could be beneficial for the treatment of malignancies. As mentioned before, the expression of SUMOylation cascade proteins, SENPs, SAE1/2, UBC9, and E3-ligases, are upregulated in multiple cancers. Inhibitors of E1 and E2 can block the SUMOylation cascade and inhibitors of SENPs can block deSUMOylation of subsets of targets and prevent the maturation of SUMOs. Blocking the SUMOylation cascade leads to impaired cell proliferation and stimulates antitumor immune responses. A significant number of natural and synthetic inhibitors targeting SAE1/2, UBC9, and SENP1, -2, and -3 in the preclinical phase have been reported. Ginkgolic acid and its structural analog, anacardic acid, were the first natural compounds identified to inhibit SUMO E1 [133]. Natural source-derived SAE inhibitors demonstrate micromolar range potency and can have several non-SUMO-related effects in cells. Development of synthetic inhibitors enabled more specific and efficient targeting of the SUMO pathway. A better understanding of cell type-specific effects of SUMO inhibitors and cancers that respond to treatment is essential to have more SUMOylation inhibitors in clinical trials. Currently, TAK-981 is the only SUMOylation inhibitor that is evaluated in clinical trials for a broad range of cancers [134].

Numerous studies have proven that NEDD8 and NEDDylation enzymes (e.g., NAE, UBE2M, UBE2F, DCN1, and NEDD8 E3 ligases) are overexpressed in human cancers, including lung cancer [135], liver cancer [136], colorectal cancer [41], intrahepatic cholangiocarcinoma [137], nasopharyngeal carcinoma [138], esophageal squamous cell carcinoma [139], and glioblastoma [140]. Cullins also play key roles in cancers. The association of increases in CUL3 and CUL4A levels with tumor progression [141] suggests the involvement of cullins in cancer. A substrate receptor protein that forms a complex with CUL1, F-Box, and WD repeat domain-containing 7 (FBW7), is mutated in 6% of all cancers and can be mutated up to 30% in some cases of leukemias or gastrointestinal cancers [142]. Furthermore, the dysregulation of many key substrate proteins of CRLs regulated by NEDD8 is associated with cancer [28]. These include: cell cycle regulators cyclin E [143], an early mitotic regulator (EMI1) [144], and p27 [143]; transcription factors NRF2 [145] and HIF-1α [146]; transcription inhibitor pIκBα [147]; and DNA regulator CDT1 [148]. Non-cullin NEDD8 substrates are also essential to cancer pathways [28], such as p53, whose function is inhibited by NEDD8 [37]. NEDD8 modifies and regulates E3 ubiquitin-protein ligase (SMURF1). NEDD8 is overexpressed with SMURF1 in colorectal cancers [149]. BCA3, which is highly expressed in breast and prostate cancer [150], is also a substrate of NEDD8 [151]. Although an increase in NEDDylation is generally correlated with cancers, there are some instances where it can be tumor-suppressive, such as the NEDDylation of transforming growth factor beta receptor 2 (TGFβRII), leading to G1/S cell cycle arrest [152].

NEDDylation regulates the tumor microenvironment (TME), which is made up of tumor cells, immune cells, cancer-associated fibroblasts (CAFs), and cancer-associated endothelial cells (CAEs), and it plays a role in tumor progression [153]. Inhibition of NEDDylation suppresses the tumor associated CAFs, CAEs, and macrophages but also suppresses the anti-tumor activity of T cells and dendritic cells [30].

Components of the NEDDylation pathway are frequently overactivated in numerous cancer types. An overactivated NEDDylation pathway leads to the dysregulation of CRL target protein degradation. MLN4924 (pevonedistat), is the first drug directed at the NEDD8-activating enzyme (NAE) to reach later-stage clinical development. MLN4924 effectively blocks cullin NEDDylation and inactivates CRLs. This results in accumulation of CRL substrates which then induce apoptosis, aging, and autophagy and prevent tumor growth [154,155]. This molecule is currently under several clinical investigations examining its anticancer effects against solid tumors and leukemia (ClinicalTrials.gov identifiers: NCT02122770). Five Phase I clinical trials have been completed and have demonstrated that MLN4924 alone or combined with chemotherapy is safe and exhibits an effective treatment effect. Some other Phase I clinical trials failed due to lack of specificity of MLN4924; some cancer cells developed resistance to MLN4924 [156]. Several Phase II clinical trials are currently recruiting patients. The most recent Phase III trial has been initiated to assess combination therapy of MLN4924 and azacitidine in patients with acute myelogenous leukemia (AML), myelodysplastic syndrome (MS), and chronic myelomonocytic leukemia (CMML). Additionally, to achieve specific inhibition of cullin NEDDylation, E2–E3 interaction inhibitors have been investigated. UBE2M–DCN1 protein–protein interaction appears to be a druggable interaction [157]. Discoveries of several DCN1 inhibitors have been reported and these inhibitors were used to study the effects of acute pharmacological inhibition of the DCN1–UBE2M interaction along the NEDD8–CUL pathway [158].

## 4. Ubiquitination, SUMOylation, and NEDDylation Assay Capabilities

### 4.1. Ubiquitination

The rapidly expanding field of ubiquitin research requires the development of novel technologies that will enable researchers to efficiently explain the physiological and pathological functions of the regulatory enzymes in this pathway. Biochemical methods exploit linkage-specific ubiquitin chains, UBDs [159], and DUBs [160] to understand the role of the ubiquitin system in health and disease to advance therapeutic research aimed at drugging the ubiquitin pathway. Linkage-specific antibodies against Met1-, Lys11-, Lys27-, Lys48-, and Lys63-linked chains as well as against Ser65-phosphorylated ubiquitin have been developed and utilized in immunoprecipitation, Western blot, and ligand binding assays [161].

**Figure 5:**
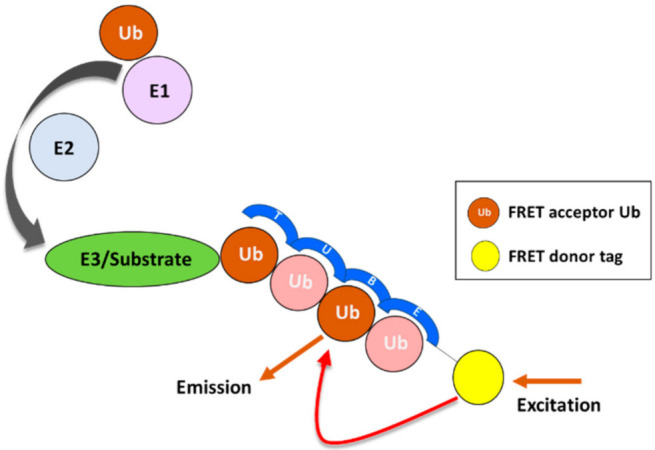
Detection of substrate ubiquitination using tandem ubiquitin-binding entities (TUBEs). Schematic representative of a FRET assay that involves FRET donor tag-labeled TUBEs that bind to polyubiquitin chains synthesized by the target E3 ligase.

Historically, the identification of ubiquitination sites in proteins has been hindered by the low stoichiometry of modification, and immunoprecipitation could be a suitable method to enrich the pool of ubiquitin-modified proteins from the total pool of proteins. A pull-down directed against (poly)ubiquitin and subsequently visualized by SDS-PAGE and Western blotting with immunostaining for the protein of interest would be the most convenient method to demonstrate direct ubiquitination of the target protein. For more accurate quantification, ligand binding assays have been developed, such as capturing the target protein and subsequently detecting the (poly)ubiquitin signal or capturing total ubiquitin and detecting specific linked chains (Met1, Lys11, Lys27, Lys48 and Lys63) [162]. In such complex ligand binding assays, the (poly)ubiquitin signals detected might be misleadingly elevated due to putative binding partners which might be ubiquitinated as well. Therefore, additional experiments are required to determine the interference. More recently, tandem ubiquitin-binding entities (TUBEs) have been developed as a substitute for antibodies in methods such as immunoprecipitation, ubiquitin proteomics, Western blotting, and ligand binding assays [163] (Figure 5).

**Figure 6:**
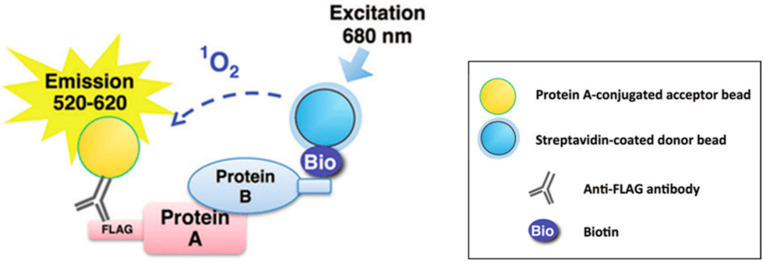
Amplified luminescent proximity homogeneous assay screen (AlphaScreen) Method. AlphaScreen allows screening for a broad range of targets. The technology provides an easy and reliable means for determining the interaction of proteins and also the effect of compounds on biomolecular interactions. AlphaScreen assay is adaptable for measuring protein activities and proteins harboring post-translational modifications.

The electrochemiluminescence (ECL) detection method has been utilized to measure the ubiquitination of target proteins, providing improved sensitivity and wider assay ranges [162,164]. Additionally, high-throughput cellular assays have also become available to study ubiquitin modifications. For instance, a high-throughput cellular assay to measure changes in MDM2 auto-ubiquitination and degradation was developed [165]. In another assay, reporter fusions, which are efficiently cleaved by ubiquitin/ubiquitin-like proteases, were utilized for quantifying activity [166]. The high-throughput assays developed to probe the ubiquitination cascade at different levels are all reconstituted systems of relevant purified proteins. Resonance energy transfer (RET) methods (e.g., fluorescence resonance energy transfer and bioluminescence resonance energy transfer) and the amplified luminescent proximity homogeneous assay screen (AlphaScreen) are some of the approaches that provide flexibility, adaptability, and sensitivity [167] (Figure 6).

The AlphaScreen is a solution-phase and homogeneous assay that does not require wash and separation steps and has greater sensitivity and wider dynamic range. In comparison to RET methods, an advantage of the AlphaScreen is that it works without the stringent short distance and orientation restrictions on the donor–acceptor pair. Recently, a fluorescence polarization assay was developed to monitor all stages of ubiquitin conjugation and deconjugation in real time. This assay enabled the development and validation of a chemical inhibitor of the E1 ubiquitin-activating enzyme, as well as assessment of the activities and specificities of E2s, E3s, and DUBs [168]. 

The vast majority of bioanalytical methods for the detection of ubiquitination primarily focus on the analysis of a single protein, either ubiquitin or a specific ubiquitinated protein. A bottom-up proteomic strategy adopts a less biased approach and can identify and quantify PTMs with a system-wide approach. Employing a bottom-up approach, products of tryptic cleavage of ubiquitinated proteins are analyzed using mass spectrometry. During trypsinization, ubiquitin is cleaved at Arg74, resulting in a di-glycine tag of 114.04 Da on the corresponding substrate proteins, enabling identification of ubiquitinated sites [169]. One of the proteomic challenges in the analysis of ubiquitin and ubiquitin-like modifiers is the low stoichiometry of ubiquitination, NEDDylation, and SUMOylation. Tag-based enrichment or antibody-based immunoprecipitation has been used to enrich the PTMs and identify the sites of modification [170]. The development of an antibody that recognizes di-glycine-modified lysines has resulted in enhanced utility for the enriched study of ubiquitinated sites through mass spectrometry. This has led to identifications of over 90,000 ubiquitinated sites [171]. In addition to the site localization of ubiquitin on the substrate protein, the architecture of ubiquitin chains can be studied using middle-down proteomics. In middle-down proteomics, restricted protein digestion is used, generating larger peptides which are identified using mass spectrometry [172]. Using this approach, native ubiquitin chains can undergo restricted trypsinolysis, specifically between Arg74 and Gly75 of ubiquitin, breaking the polyubiquitin chain into individual ubiquitins with or without the di-glycine motif. If the individual ubiquitin has two di-glycine tags, then the polyubiquitin chain is branched [173]. Ubiquitin clipping is a similar method to understand the signals and architecture of polyubiquitin chains wherein viral protease lBpro is used to remove ubiquitin from the substrate leaving the di-glycine tag attached to the modified residue instead of restricted trypsinization. Quantification of polyubiquitin is possible by the identification of multiple di-glycine-modified branch-point ubiquitin [174]. Further advancements in mass spectrometry instrumentation are required to completely understand the architecture of polyubiquitin chains.

### 4.2. SUMOylation

Although more challenging due to its low abundance, SUMOylation has also been extensively studied using similar approaches. To analyze endogenous SUMO conjugates, a method called protease-reliance identification of SUMO modification (PRISM) has been developed for the identification of SUMOylation sites based on the chemical blockade of all free lysines and treatment with a SUMO-specific protease. Subsequently ‘freed’ lysines are biotin-tagged and identified by high-resolution mass spectrometry [175].

Different single or double epitope tags fused to the N-terminus of SUMO have been used to enrich and identify mono-, multi-, or poly-SUMOylated proteins. Examples include 6x–10xHis, FLAG, MYC, 6xHis–FLAG, 6xHis–HA, FLAG–TEV, and ProtA–TEV–CBP for use in cell culture as well as transgenic organisms [176]. DeSUMOylating activity is high in cell lysates, so chemical inhibitors or ideally denaturing conditions are used during capture of conjugates to preserve SUMOylation [177]. The in vitro SUMOylation assays evaluate the ability of purified UBC9 to catalyze SUMOylation of substrate proteins. Since usually only a small fraction of the total substrate protein is SUMO-modified, SUMOylation, in most cases, can only be detected in the presence of mutated SUMO E3 ligase with increased ligase activity. Immunoprecipitation of either an endogenous or a transfected and epitope-tagged protein of interest from cell lysates followed by running a Western blot using anti-SUMO antibodies has been one of the most commonly used approaches [20]. Alternatively, SUMOylation of a purified recombinant protein of interest or ^35^S-labeled translation product, reconstituted in an in vitro SUMOylation enzymatic reaction, can be detected using Western blotting or autoradiography, respectively [20]. Similar to ubiquitination, SUMOylation can also be monitored in real time using either a fluorophore-tagged substrate or SUMO protein in a SUMOylation reaction [178,179]. A FRET-based assay was developed using yellow fluorescent protein (YFP)-tagged mature SUMO-1 and cyan fluorescent protein (CFP)-tagged Ran GTPase-activating protein 1 (RanGAP1) and its utility has been demonstrated in a high-throughput screening effort. Most recently, a new technology for the screening of novel SUMOylation inhibitors as well as for genome-wide identification of novel SUMOylated proteins was developed to detect SUMOylation of RanGAP1 with SUMO-1 and the interaction between SUMO-1 and -3 and a previously reported SUMO-binding motif (SBM). The method does not require any protein chemical modification and the signal is strictly dependent on the presence of E1, UBC9 (E2), ATP, SUMO-1, and RanGAP1, confirming the specificity of the reaction [167].

To study SUMOylation using proteomics tools, an enzyme wild-type alpha-lytic protease (WaLP) has been used to cleave threonine, generating di-glycine remnant peptides, which can be enriched using a di-glycine antibody [180]. Similarly, with the use of other enzymes, such as Lys-C and Asp-N digestion followed by immunoprecipitation, 14,869 SUMO2/3 sites have been identified [181]. Another approach for generating a di-glycine tag for SUMOylated peptides involves introducing mutations SUMO1 T95R and SUMO2 T91R to generate smaller tags, resulting in a di-glycine motif that is similar to ubiquitin [182].

### 4.3. NEDDylation

Traditional approaches to studying the NEDDylation of cullins utilize antibodies which can be nonspecific and limit measurements of end-point assays. In order to monitor the covalent NEDDylation of cullin 1, the NanoLuc^®^ Binary Technology (NanoBiT), which is a structural complementation reporter system composed of a Large BiT (LgBiT; 18kDa) subunit and a small complimentary peptide, was developed for real-time monitoring of protein–protein interaction dynamics [183]. When the two proteins interact, the subunits come together to form an active enzyme and generate a bright luminescent signal in the presence of substrate. Other strategies to verify whether a protein of interest is a substrate of NEDDylation include a pull-down of histidine-tagged NEDD8 for in vitro reactions or co-immunoprecipitation of the protein of interest for cell-based experiments followed by Western blot analysis of NEDD8 [184,185]. Several of the assays involved in NEDDylation studies aim to investigate the mechanistic impact on alterations to the NEDDylation pathway. A common method used to analyze these effects in lysates is to fractionate by SDS-PAGE and blot for members of the NEDDylation pathway, such as the E2s and CRL substrates [186]. Different variations of the fluorescence resonance energy transfer (FRET) assay, such as time-resolved FRET (TR-FRET) and homogenous time-resolved fluorescence (HTRF), can monitor specific interactions, including NEDD8 ligation by analysis of DCN1–UBE2M interaction [36] and NAE activity by ABP1 probe [187] or analyzing NEDD8–E2 interaction [188]. AlphaScreen has also been used to measure the interaction between NEDD8 and E2 [189] or NEDD8 and NAE [190]. Cell-based assays, such as the cellular thermal shift assay (CETSA), isothermal titration calorimetry (ITC) [36], biolayer interferometry [191], and ATP competitive inhibiting detection [192,193], have also proved to be useful methods to study NEDDylation and evaluate the efficacy of potential NEDDylation inhibitors. Mass spectrometry offers a powerful tool for the biochemical complexity and functional diversity of the NEDDylation system similar to the ubiquitin system. One of the biggest challenges, though, in identification of NEDD8 conjugation sites is that the C-terminal sequence identity of ubiquitin (–LRGG) results in the same di-glycine remnant on modified lysine residues upon trypsin digestion as NEDD8; therefore, the method cannot discriminate between the two modifications. By using mutations, tags, and antibodies, ubiquitin-like modifiers can now be identified with better results in mass spectrometry [194]. For instance, a new technique of NEDD8–ubiquitin substrate profiling (sNUSP) has overcome this challenge and allowed discrimination and quantitation of NEDD8-modified versus ubiquitin-modified peptides [195]. This method is based on the substitution of Arg74 of NEDD8 to lysine (R74K) via CRISPR–Cas9 editing of the endogenous NEDD8 gene in cultured cells. Upon Lys-C digestion (in place of trypsin), K-εGG peptides are generated specifically from NEDD8R74K-modified substrates. NEDD8R74K-specific K-εGG profiling identified a large set of NEDDylation site substrate proteins [195].

## 5. Conclusions

Our understanding of ubiquitination, from its being initially defined as having a function to degrade proteins to now being considered the most versatile protein-modification system that impacts virtually all realms of the life sciences, has significantly advanced. The study of ubiquitination and deubiquitination, essential regulators of metabolic reprogramming in cancer cells, will continue to advance for development of specific, more efficient, and less toxic drugs that disrupt or enhance specific interactions between substrates and E3 ligases or DUBs. The main contribution of SUMOylation and NEDDylation in protein homeostasis is their complex interplay with ubiquitin. As central players in cell homeostasis, ubiquitination, SUMOylation, and NEDDylation are involved in the control of most cellular processes and thus have a critical role in health and disease, in drug response, and eventually in disease prognosis. There is a constant effort to develop novel and sensitive PTM identification, characterization, and quantification techniques. The motivation for investing in these techniques is considerable because of the direct connection that these signals have to human health and disease. The development of strategies to characterize the interplay (e.g., antagonism and sequential modification) between ubiquitination, SUMOylation, and NEDDylation pathways will facilitate progress in advancing novel cancer therapeutic strategies.
